# Cavernous hemangioma of the mediastinum originating from a left persistent superior vena cava

**DOI:** 10.1016/j.xjon.2025.05.007

**Published:** 2025-05-23

**Authors:** Mo'men Alashwas, Wedad Alashwas, Ahmad Dalal, Natalie Khamashta, Hamad Madi

**Affiliations:** a Faculty of Medicine, Al-Quds University, Abu Dis, Jerusalem, Palestine; b Department of Cardiothoracic Surgery, Palestine Medical Complex, Ramallah, West Bank, Palestine

**Figure:**
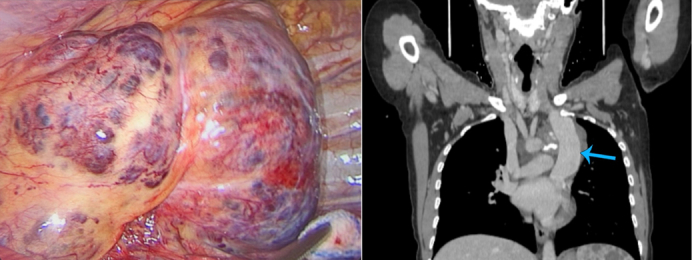
The highly vascular cavernous hemangioma and CT imaging of the dilated left PSVC.

**Central Message**

Left persistent superior vena cava is a very unusual site of origin of mediastinal hemangiomas. Preoperative diagnosis can be challenging. Proximity to critical structures complicates management.

A 31-year-old female patient presented with a complaint of shortness of breath on exertion over the past year. The patient has a nonsmoking history and no significant medical history (Video 1). The institutional review board or equivalent ethics committee of Al-Quds University did not approve this study because according to the local and institutional guidelines, institutional review board approval is not required for case reports. The subject(s) provided informed written consent for the publication of the study data.

Upon further investigation, computed tomography (CT) imaging of the chest revealed a soft-tissue mass measuring 10 × 11 cm in the left mediastinum (Figure 1). It also revealed the presence of a left persistent superior vena cava (PSVC), which was engulfed by the mass along with the left subclavian artery, in addition to being in direct contact with the aortic arch. The spleen was also enlarged measuring up to 13.5 cm.

**Figure 1:**
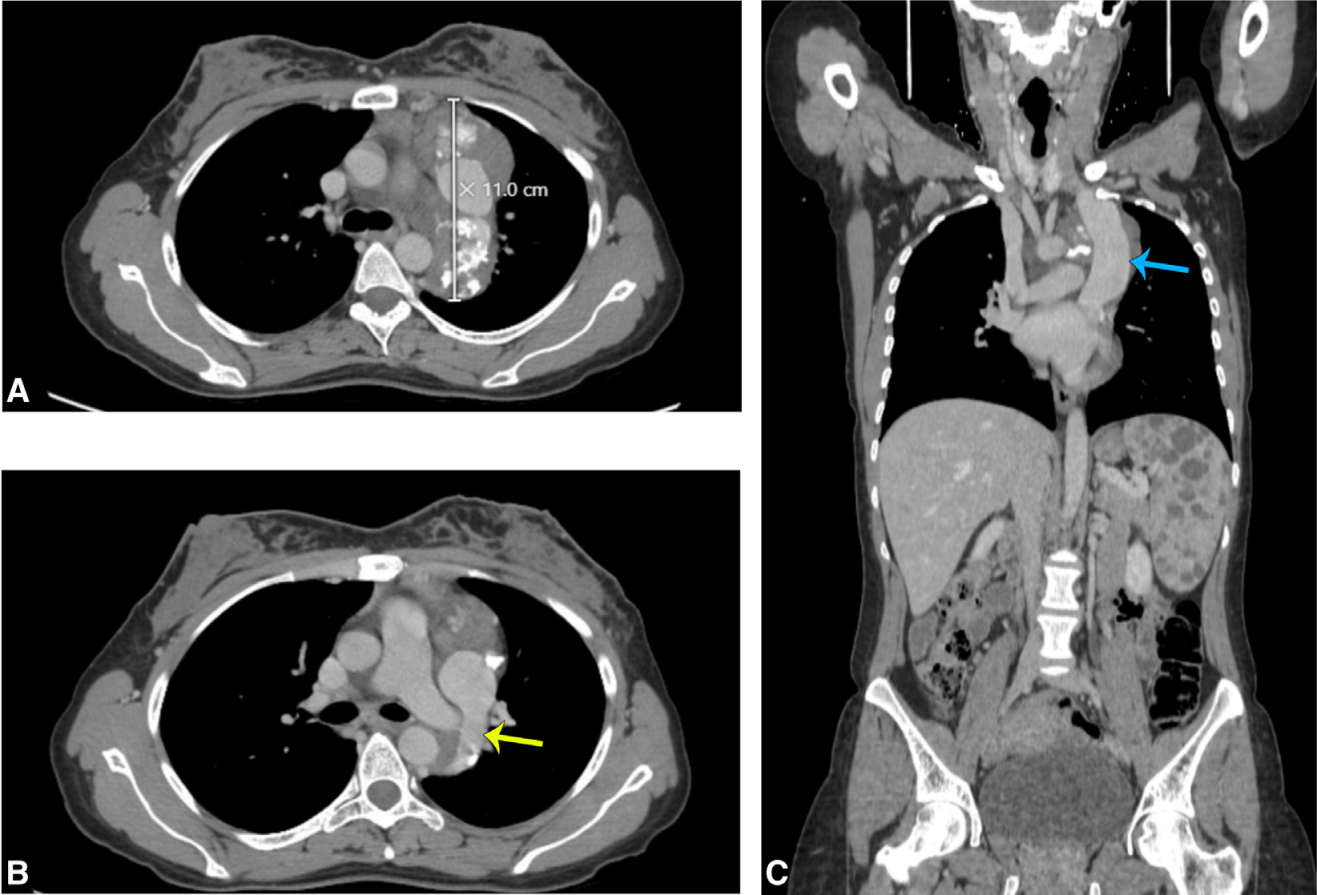
Venous phase of preoperative computed tomography scan. A, The lesion at its widest extent. B, The main feeding vessel of the hemangioma originating from the left superior vena cava (*yellow arrow*). C, Sagittal view showing the dilated left superior vena cava (*blue arrow*). Diffuse lesions of the spleen can be seen.

Positron emission tomography-CT was performed, which revealed a bulky mediastinal mildly hypermetabolic soft-tissue mass with associated lymphadenopathy. In addition, diffuse photopenic lesions of the spleen were noted.

CT-guided biopsy was attempted twice; however, both times only necrotic tissue was retrieved and no diagnosis was made. Endobronchial ultrasound was performed, and no abnormal findings were detected. The patient was then referred for exploratory video-assisted thoracoscopic surgery (VATS).

Exploratory VATS showed a vascular lesion extending from the left superior sulcus into the inferior phrenic vein. Anteriorly, it involved the left phrenic nerve and completely masked the aortic arch and arch vessels with no solid component seen. The lesion was considered to be compatible with a large hemangioma. No resection was attempted during the procedure because of the highly vascular appearance of the lesion.

Intraoperatively, a 10-cm anterolateral thoracotomy incision was done. The lesion was observed in the anterior mediastinum extending posteriorly, it was highly vascularized with multiple sacs (Figure 2). The mass engulfed the aortic arch with all its branches, dissection was done carefully using a dissector and energy device. Shaving of the tumor away from the innominate vein and the brachiocephalic artery was done. We then moved posteriorly as the tumor was adherent to the descending aorta, dissection was done the same way. A feeding vessel was noticed along the vertebral column, which was secured and cut. The left vena cava was visualized, which was huge, aneurysmatic, and engulfed within the tumor. Dissection was done carefully until we found a huge tributary that was feeding the tumor, and it was secured and cut. Dissection was performed over the left lung hilum to separate the tumor. During dissection, the left phrenic nerve was identified entering the tumor, so we sacrificed it for optimal tumor dissection. Part of the tumor was left at the level of the left subclavian artery and the second sac of persistent left vena cava because of the high risk of bleeding, this part was marked with clips to guide oncologic follow-up. Postoperatively, the drains were removed on the second day and she underwent physiotherapy and respiratory therapy. The patient was discharged on the fourth day in a very well condition.

**Figure 2:**
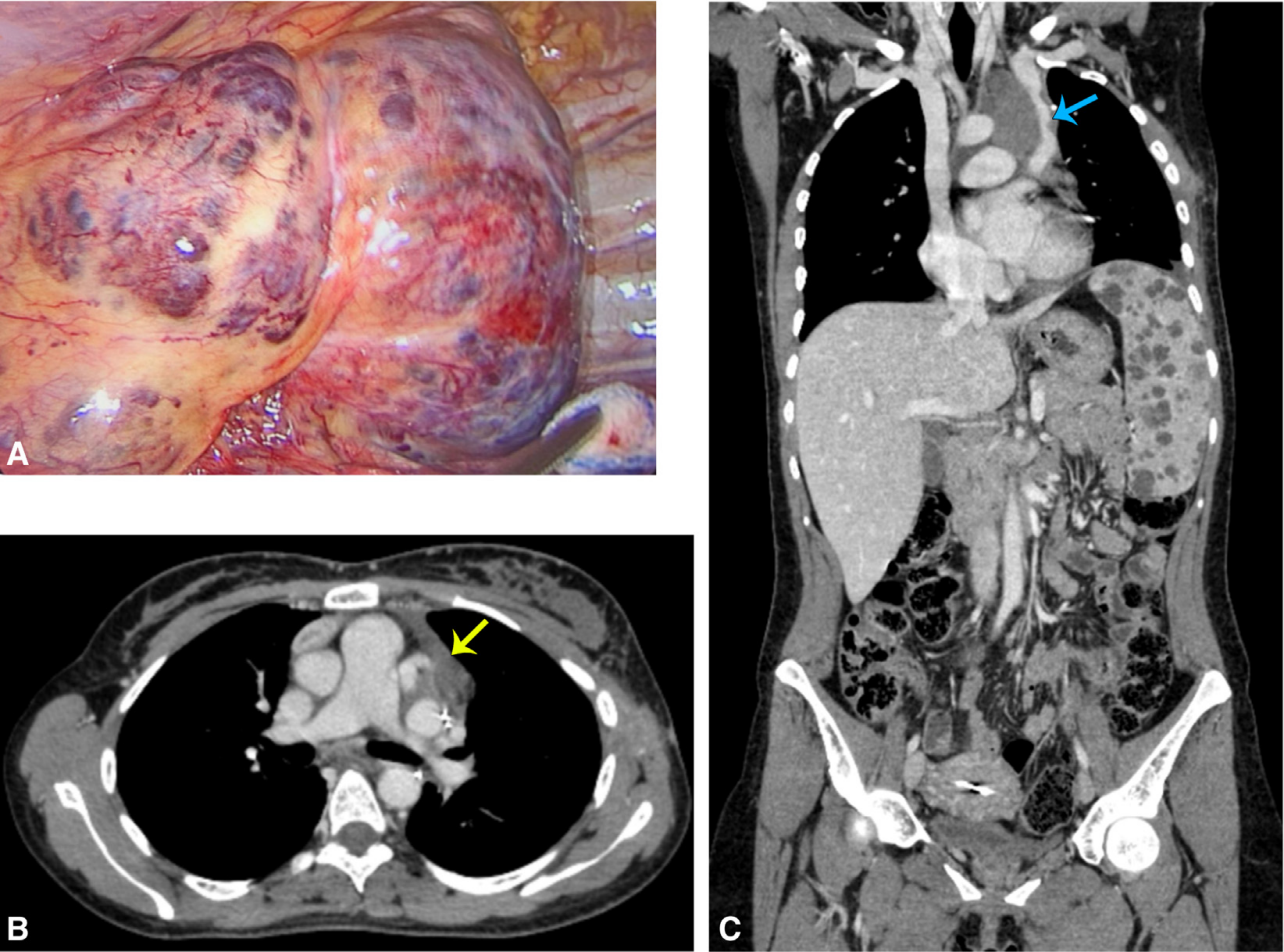
A, Intraoperative image showing the vascular surface of the lesion. B, Venous phase of postoperative computed tomography scan showing the remnants of the resected lesion (*yellow arrow*). C, Sagittal view showing the left superior vena cava postoperatively (*blue arrow*).

Histopathologic analysis of 3 resected pieces of the mass, measuring 8 × 8 × 2 cm, and 3 × 2 × 1 cm, respectively, was done. The cut surfaces were hemorrhagic. Microscopically, the sections showed variably sized, and ectatic/dilated vascular channels lined by a single layer of endothelial cells, in addition to lymphocytic infiltration. Histologic diagnosis of cavernous hemangioma was made, and no evidence of malignancy was noted in the examined samples.

## Discussion

Mediastinal hemangiomas (MHs) represent uncommon congenital vascular anomalies that usually present in adults around the age of 35 years with the anterior mediastinum representing the most common location. Mediastinal hemangiomas are categorized according to the size of their vascular spaces into 3 subtypes, capillary, venous, and as in our case cavernous. Capillary and cavernous hemangiomas represent the vast majority of these tumors.^1^^,^^2^

Mediastinal hemangiomas can easily be mistaken for other more common mediastinal masses and malignancies on preoperative evaluation and imaging studies; therefore, histopathologic confirmation of mediastinal hemangiomas after resection is essential.^3^ In our case, all preoperative imaging was inconclusive, with exploratory VATS raising the highest suspicion. Diagnostic confirmation of cavernous hemangioma was attained only after surgical resection.

On imaging, both magnetic resonance imaging (MRI) and CT imaging are able to demonstrate the heterogeneity of MH. MRI is generally more sensitive for its components, such as thrombosed and nonthrombosed vessels, fibrosis, hemorrhage, and calcifications, among others. Thrombosis can be noted on both CT and MRI and is a relatively specific finding but is not sensitive. MH can present with various enhancement patterns on contrast CT, ranging from central, mixed, and peripheral to nonspecific. Delayed enhancement, especially with a gradual centripetal filling pattern, is most suggestive. MRI is more sensitive for delayed enhancement. Large aberrant veins that drain the lesion are specific for MH and may indicate the current or past presence of high blood flow within the lesion. Those veins are essential to consider when planning the surgery, because they may pose a significant risk of bleeding.^3^

Some associated manifestations can be present in patients with MH, such as kidney, liver, and splenic hemangiomas, which might explain the mild splenomegaly noted in our patient's case.^3^

Left PSVC is a rare vascular anomaly but is the most common among thoracic venous anomalies. It has a greater prevalence among patients with congenital heart disease, ranging from 1.3% to 11% compared with 0.2% to 3% in the general population. It is usually an incidental finding. The presence of clinical manifestations is influenced by drainage site and concurrent abnormalities.^4^

**Figure 3:**
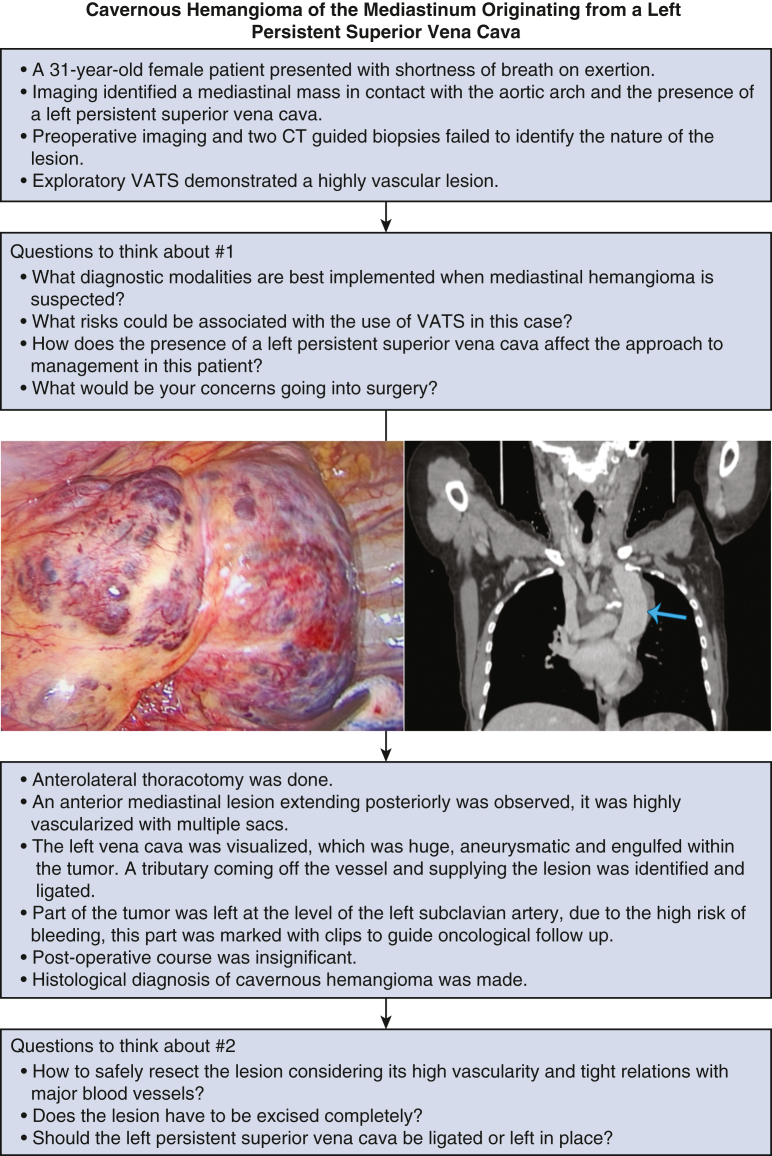
Questions and interesting points in the diagnosis and management of patients with rare mediastinal hemangiomas. *Blue arrow* indicates the dilated left superior vena cava preoperatively. *CT*, Computed tomography; *VATS*, video-assisted thoracoscopic surgery.

Upon review of the literature, only one case was found describing the incidence of a cavernous hemangioma in association with a persistent left superior vena cava. In this case, a communicating vein was identified connecting the lesion to the coronary sinus, with the lesion involving most of the right atrial wall and a section of the interatrial groove. Blood flow was not reported inside the mass, which measured 11 × 6.5 × 4.5 cm. The left PSVC was ligated during surgery.^5^ Our case was special in that the heart was not involved in the lesion and no cardiac attachment was identified. The lesion was also highly vascular with significant blood flow inside. In addition, during management feeding vessels originating from the left PSVC were identified and ligated but the left PSVC was left intact. Further questions concerning this case should also be addressed (Figure 3).

## Conflict of Interest Statement

The authors reported no conflicts of interest.

The *Journal* policy requires editors and reviewers to disclose conflicts of interest and to decline handling or reviewing manuscripts for which they may have a conflict of interest. The editors and reviewers of this article have no conflicts of interest.

## Supplementary Data

Video 1 A rare case of cavernous hemangioma presenting in a left persistent superior vena cava. Video available at: https://www.jtcvs.org/article/S2666-2736(25)00160-3/fulltext.
