# Supplementary figures and images for: Cavernous hemangioma of the mediastinum originating from a left persistent superior vena cava
Source: JTCVS Open. 2025 May 23;26:288–91. doi: 10.1016/j.xjon.2025.05.007 (PMC12414399; doi:10.1016/j.xjon.2025.05.007)

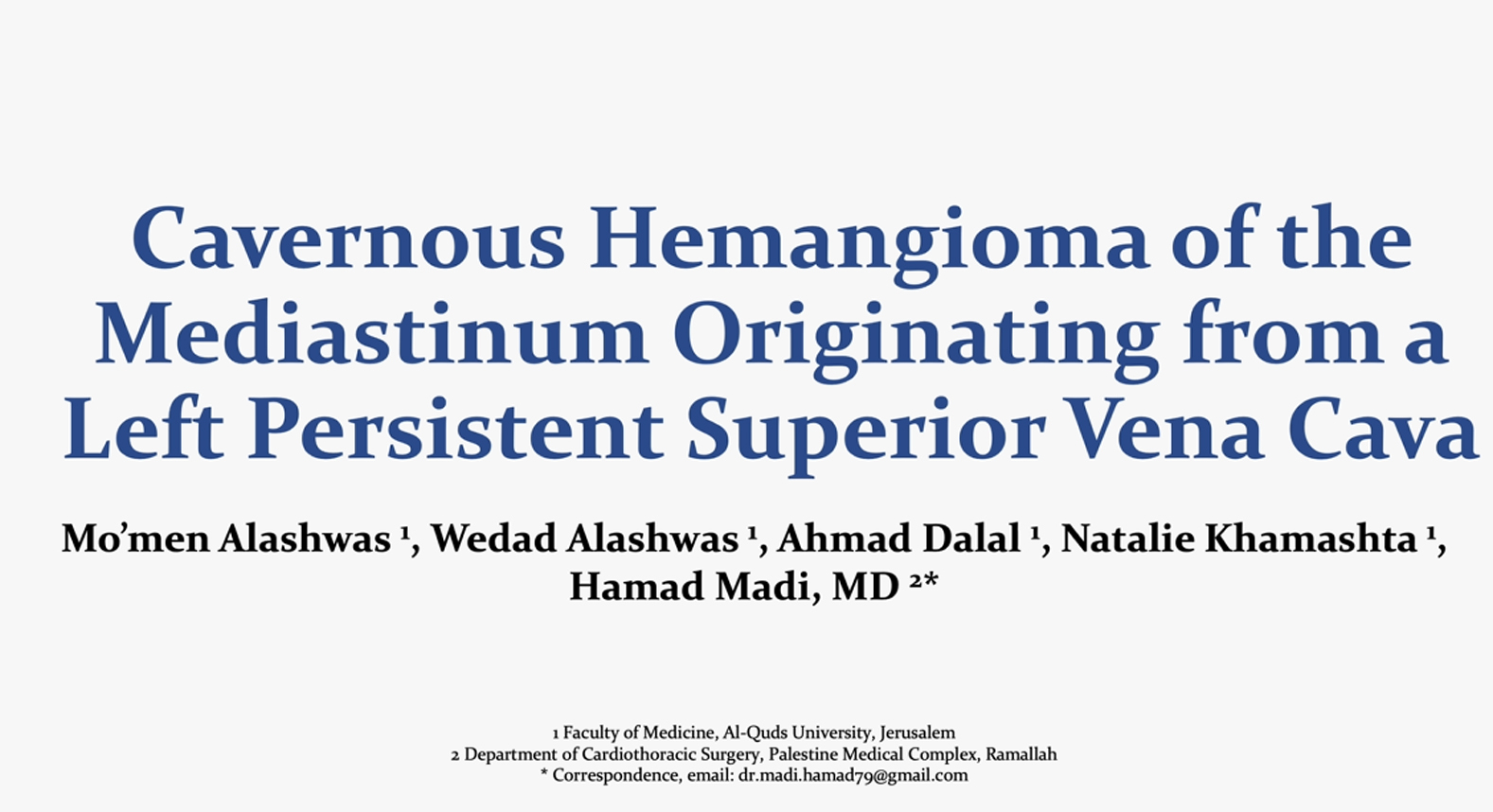

Supplement: Video 1 — A rare case of cavernous hemangioma presenting in a left persistent superior vena cava. Video available at: https://www.jtcvs.org/article/S2666-2736(25)00160-3/fulltext. [file fx2.jpg]
